# Specialization of amygdala subregions in emotion processing

**DOI:** 10.1002/hbm.26673

**Published:** 2024-04-09

**Authors:** Izelle Labuschagne, Juan F. Dominguez, Sally Grace, Simone Mizzi, Julie D. Henry, Craig Peters, Christine A. Rabinak, Erin Sinclair, Valentina Lorenzetti, Gill Terrett, Peter G. Rendell, Mangor Pedersen, Darren R. Hocking, Markus Heinrichs

**Affiliations:** ^1^ Healthy Brain and Mind Research Centre, School of Behavioural and Health Sciences Australian Catholic University Melbourne Victoria Australia; ^2^ School of Psychology The University of Queensland Brisbane Queensland Australia; ^3^ School of Psychology Deakin University Melbourne Victoria Australia; ^4^ School of Health and Biomedical Science RMIT University Melbourne Victoria Australia; ^5^ Department of Pharmacy Practice Wayne State University Detroit Michigan USA; ^6^ Department of Psychology and Neuroscience Auckland University of Technology Auckland New Zealand; ^7^ The Florey Institute of Neuroscience and Mental Health The University of Melbourne Melbourne Victoria Australia; ^8^ Institute for Health & Sport Victoria University Melbourne Victoria Australia; ^9^ Department of Psychology Albert‐Ludwigs‐University of Freiburg Freiburg im Breisgau Germany; ^10^ Freiburg Brain Imaging Center University Medical Center, Albert‐Ludwigs University of Freiburg Freiburg im Breisgau Germany

**Keywords:** centromedial, facial expressions, fear, fMRI, superficial

## Abstract

The amygdala is important for human fear processing. However, recent research has failed to reveal specificity, with evidence that the amygdala also responds to other emotions. A more nuanced understanding of the amygdala's role in emotion processing, particularly relating to fear, is needed given the importance of effective emotional functioning for everyday function and mental health. We studied 86 healthy participants (44 females), aged 18–49 (mean 26.12 ± 6.6) years, who underwent multiband functional magnetic resonance imaging. We specifically examined the reactivity of four amygdala subregions (using regions of interest analysis) and related brain connectivity networks (using generalized psycho‐physiological interaction) to fear, angry, and happy facial stimuli using an emotional face‐matching task. All amygdala subregions responded to all stimuli (*p*‐FDR < .05), with this reactivity strongly driven by the superficial and centromedial amygdala (*p*‐FDR < .001). Yet amygdala subregions selectively showed strong functional connectivity with other occipitotemporal and inferior frontal brain regions with particular sensitivity to fear recognition and strongly driven by the basolateral amygdala (*p*‐FDR < .05). These findings suggest that amygdala specialization to fear may not be reflected in its local activity but in its connectivity with other brain regions within a specific face‐processing network.

## Graphical abstract

Using functional magnetic resonance imaging, we show that the amygdala should be viewed as a group of heterogeneous subregions when processing emotional stimuli, with specialization of fear only evident in the connectivity patterns of the amygdala subregions with the rest of the brain.

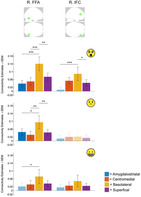

## 1 INTRODUCTION

Animal and human studies have demonstrated that the amygdala plays a prominent role in the rapid detection and recognition of information in the environment that is critical for survival (Le Doux, 2012). In particular, the amygdala has been shown in many human studies to have a robust response to negative or threatening stimuli, such as fearful faces (Adolphs, 2008; Le Doux, 2003). An association between the amygdala and fear is also supported by findings showing that many prevalent mental health disorders, including anxiety disorders, autism, and severe substance use disorders, have been linked to dysfunctional amygdala responses, most notably relating to fear processing (Gilpin et al., 2015; Herrington et al., 2016).

However, the view that the amygdala specializes in fear processing has been challenged (Janak & Tye, 2015), with evidence suggesting a role for the amygdala in the processing of a range of emotions in addition to fear (Diano et al., 2017; Phelps & LeDoux, 2005; Sergerie et al., 2008). This new perspective has potentially important implications for our understanding of the amygdala's role in emotional functioning, which underpins everyday socio‐emotional behaviors and mental health. Therefore, a clearer and more nuanced understanding of the intricacies of the amygdala circuitry response to emotional stimuli is needed.

Consideration of the amygdala at the subregional level provides a compelling way to address this question, first by investigating the extent to which different subregions respond differently to emotional stimuli, and second, through investigation of amygdala subregional connectivity with wider brain networks. Human studies that have previously tested whether there are different subregional amygdala responses to emotional stimuli appear to support the view that the amygdala is less specialized for processing fearful stimuli than previously thought. For example, robust and significant activations specifically of the basolateral, centromedial, and superficial amygdala subregions have been identified in response to socially relevant fearful, happy, and neutral faces (relative to non‐social stimuli such as houses), with the superficial and centromedial amygdala showing particularly robust activations compared to the other subregions (Goossens et al., 2009; Hrybouski et al., 2016; Hurlemann et al., 2008). However, conclusions from these studies are limited owing to their sample sizes (ranging from 14 to 25 participants) and the fact that they mostly used standard‐resolution functional magnetic resonance imaging (fMRI) acquisition parameters (e.g., echo planar imaging) that typically lack the resolution to clearly capture the grey matter boundaries of the subregions, and thus highlighting the need for more work to be done before drawing conclusions.

In addition, as previously noted, another important way of understanding the amygdala's role in emotion processing, particularly in relation to its potentially heightened sensitivity to fear, is through consideration of its links to wider brain networks. Research has demonstrated that the whole amygdala is functionally connected to different brain regions depending on the type of emotion being processed. This is despite similarities in the activation patterns of the whole amygdala in response to these same emotions (Diano et al., 2017). However at the subregional level, while evidence for differential connectivity effects has been reported during resting‐state fMRI paradigms (Kerestes et al., 2017; Qin et al., 2014), to the best of our knowledge, this has only been examined using task‐based fMRI in school‐aged children to explore differences in connectivity patterns in response to negative emotions specifically (Tian et al., 2021). Tian et al. (2021) identified alterations in connectivity between the basolateral/centromedial amygdala subregions and prefrontal regions (i.e., medial, dorsolateral and ventromedial prefrontal cortex) when processing negative emotional faces (compared to shapes) in a sample of typically developing school‐aged children. However, they did not explore whether connectivity patterns differed based on the type of emotional stimuli (e.g., fearful vs. happy OR negative vs. positive).

Additionally, consideration of the impacts of sex and laterality on amygdala activation to emotional stimuli is important to gain a more nuanced understanding of the amygdala's role in emotion processing. Evidence from a meta‐analysis demonstrates that females have greater left amygdala activation in response to negative emotional stimuli, and males have greater left amygdala activation in response to positive emotional stimuli (Stevens & Hamann, 2012). Furthermore, evidence from a separate meta‐analysis identified greater left (vs. right) amygdala activity during negative facial emotion processing (Fusar‐Poli et al., 2009). While there is preliminary evidence of differences in laterality in the centromedial and basolateral subregions (Tian et al., 2021), there is limited research investigating the effects of sex and laterality at the subregional level.

This exploratory study was designed to provide the most rigorous test to date of (i) the extent to which different subregions respond differently to emotional stimuli, and (ii) the first test of whether amygdala subregional connectivity with broader brain networks differs when processing fear relative to other emotions. Additionally, this study aimed to explore whether amygdala subregional findings differed from whole amygdala finding and whether there was a significant effect of sex and laterality on the local activation to emotional expressions (vs. Shapes) of the amygdala and its subregions. To achieve these aims, the increased spatial resolution and temporal sampling of multiband brain imaging was used to not only tease apart differences in the localized activity of the amygdala and its subregions, but also to map the connectivity of the amygdala and its subregions to the broader brain networks. To ensure sufficient power, a large sample of 86 healthy adults (44 females) completed an emotional face‐matching task known to reliably activate the amygdala and related brain networks during multiband magnetic resonance imaging (MRI; Hariri et al., 2002).

## 2 METHODS

### 2.1 Participants

We recruited 100 right‐handed participants with the following exclusion criteria: left‐handed, history of or current psychiatric/neurological disorder, English as a second language, taking psychotropic medication, head trauma (unconscious for >5 min), substance abuse including smoking, and usual MRI contraindications including the presence of metal objects in the body that cannot be removed or made safe for MRI. Fourteen participants were excluded for various reasons. Three participants withdrew from the study, three had incomplete scans due to failing to complete the task, and two had failed acquisition of MRI data. Six participants were excluded due to excessive motion during the scan. Among these, one was excluded following visual quality control while the remaining five were excluded due to exceeding the threshold of having any run with an average relative root mean squared (RMS) displacement greater than 0.2 mm. Thus, the final sample comprised a total of 86 participants. The study was approved by the Human Research Ethics Committee at the Australian Catholic University.

### 2.2 Emotional face matching task

During fMRI acquisition, participants completed an emotional face‐matching task (EFMT; see Figure S1). This task elicited activation in areas, including the amygdala (see Figure S2), consistent with previous studies involving both clinical and healthy populations (Hariri et al., 2002; Labuschagne et al., 2010; Tessitore et al., 2002). There were a total of 26 experimental blocks consisting of 12 emotional face‐matching blocks (four blocks for each target emotion) and 14 shape‐matching blocks, counterbalanced across two experimental runs. Each block contained four sequential matching trials presented for 5 s. Participants used their right hand to press one of two buttons on an MRI‐compatible button box. Button‐press responses were recorded and analyzed for accuracy for each emotion condition. Presentation software (http://neurobs.com) was used to display the stimuli and record participant responses.

### 2.3 fMRI methods

Structural and functional data were acquired on a Siemens MAGNETOM Tim Trio 3.0 T Scanner with a Siemens 12‐channel Head Matrix Coil (Erlangen, Germany) at Swinburne University, Australia. A multiband echo‐planar imaging sequence was used to acquire functional images (repetition time [TR] = 1.02 s; echo time [TE] = 30 ms; flip angle [FA] = 65°; multiband acceleration factor [MB] = 5; 65 transversal slices with 96 × 96 voxels at 2 mm in‐plane resolution; 294 volumes per each of two runs). To correct geometric distortions commonly present in functional images, echo‐planar imaging data were also collected with reversed phase‐encode blips, resulting in pairs of images with distortions going in opposite directions. A T_1_‐weighted sagittal MP‐RAGE structural image was also obtained for anatomical reference (TR = 1.9 s, TE = 2.52 ms, FA = 9°, 176 slices with 1 mm*1 mm*1 mm voxels). Five participants had cropping at the top of the brain or the temporal lobes. These participants were included in the analysis as the data of interest were not located in these areas.

### 2.4 fMRI data pre‐processing

The imaging data were pre‐processed and analyzed using FSL (Jenkinson et al., 2012) and SPM12 software (Welcome Trust Centre for Neuroimaging, London, UK). The bottom slices for each functional image and the field‐maps were dropped to change the dimensions of the images from 96 × 96 × 65 voxels to 96 × 96 × 64 voxels using MRtrix. SPM12 (running on MATLAB R2019b, The Mathworks, Inc.) was used to perform slice‐timing correction and realignment of functional time series to correct for motion. FSL's TOP‐UP was then used to create a combined distortion field‐map from the phase‐encode‐reversed image pair (Andersson et al., 2003) with the result applied to the functional images with FSL's FUGUE to correct geometric distortion (Jenkinson et al., 2004). Co‐registration of anatomical and functional images, and segmentation of the *T*_1_‐weighted images were then performed through SPM12's VBM to produce grey/white matter maps for normalization via DARTEL warping (Ashburner, 2007), which was then applied to the functional images. After normalization, images were re‐sliced to 2 × 2 × 2 dimension voxels, Gaussian smoothed with a 6 mm kernel, and high‐pass filtered (0.008 Hz). No differences in the findings were observed when re‐analyzing data after applying a 3 mm smoothing kernel (see Figure S3). The anatomically defined amygdala and its subregions were generated for each hemisphere using the Anatomy Toolbox (Version 2.1) A manual visual inspection was carried out to ensure that the transformation to template space and alignment of the amygdala and its subregions were correct.

### 2.5 Statistical analysis

#### 2.5.1 Emotional face‐matching task analysis

A mixed effects model was used to investigate a main effect of condition (differences between Fear, Angry, Happy, and Shapes) on the accuracy of responses to the EFMT. Post‐hoc tests were then used to ascertain specific differences between all conditions. Sex and Age were included as covariates of no interest. Results were corrected for multiple comparisons using the false discovery rate (FDR) at *p*‐FDR < .05. As we found evidence of departures from normality, and the presence of a limited number of outliers, we implemented bootstrapping (with 2000 repetitions) in our analyses. These analyses were performed with Stata 16SE (StataCorp, 2019).

#### 2.5.2 ROI activation analysis

A general linear model was used to estimate the effects of each condition (fearful faces–Fear; angry faces–Anger; happy faces–Happy; and shapes–Shapes) at each voxel. To account for motion‐related effects in activity, the six movement parameters obtained during realignment were included in the model as regressors, along with their derivatives and the quadratic terms of both the motion and motion derivatives. Outlier volumes were also identified (using frame displacement and DVARS metrics as estimated with fs1_motion_outliers) and included as regressors in the model. The mean activation within each ROI for the contrasts Fear > Shapes, Anger > Shapes, and Happy > Shapes, was then extracted for each participant using the MarsBaR toolbox for SPM (Brett et al., 2006).

We first used repeated measures mixed effects models to investigate the effect of Emotion (differences in the BOLD response between Fear, Angry and Happy) separately on the amygdala and each subregion (i.e., the amygdalostriatal, basolateral, centromedial, and superficial subregions), together with sex, laterality, and interaction effects. A separate series of repeated measures mixed effects models were then used to evaluate the effect of region (differences between amygdala subregions responses), for each emotion separately, again, with sex, laterality and interaction effects. Planned contrasts were also implemented to evaluate if the BOLD response in the amygdala and its subregions were significantly different from 0 (i.e., different from Shapes) across Fear, Anger and Happy conditions. Age was included as covariate of no interest in all analyses. Results were corrected for multiple comparisons at *p*‐FDR < .05. As we found evidence of departures from normality (with distributions of residuals generally leptokurtic), heteroskedasticity, and a limited number of outliers, we implemented bootstrapping (with 2000 repetitions) in our analyses. These analyses were performed with Stata 16SE (StataCorp, 2019).

#### 2.5.3 gPPI connectivity analysis

The CONN toolbox (Version 18b; Whitfield‐Gabrieli & Nieto‐Castanon, 2012) was used to perform generalized psycho‐physiological interaction (gPPI) connectivity analysis. Task conditions (Fear, Anger, Happy, and Shapes) and movement parameters were imported into the CONN‐Toolbox, together with pre‐processed functional and anatomical images for each participant. Noise correction was performed using the anatomical component‐based noise correction (aCompCor) method as implemented in the CONN Toolbox (Behzadi et al., 2007). aCompCor uses white‐matter and cerebrospinal fluid masks (generated via segmentation of anatomical images) to extract principal components from the respective time series for each participant. These components, together with motion parameters and outlier volumes derived during SPM pre‐processing, were added as confounds in the denoising step of the CONN toolbox.

The gPPI analysis evaluates, for each participant, the task‐modulated connectivity between a seed region (the amygdala and its subregions) and the whole brain or other ROIs using a multiple regression model incorporating task effects, the timeseries from the amygdala and its subregions, and an interaction term (PPI term) between these two components. In this way, in the present study, the effects of the Fear, Anger, and Happy conditions on effective connectivity (relative to Shapes) were estimated and compared, and subsequently submitted for analysis at the group level, where we modeled the Emotion and Regions effects, as well as the effects of sex and laterality, with Age included as a covariate of no interest. Primary results for functional connectivity analyses are reported irrespective of hemisphere (i.e., averaging across hemispheres). In addition, we also report the effect of laterality.

First, we estimated the whole‐brain, voxel‐wise amygdala connectivity averaged across emotions to identify the amygdala network common to emotional face processing. Results for this analysis were cluster‐corrected at a family‐wise error rate (FWER) of *p‐*FWE < .05, after applying a cluster‐forming threshold of *p* = .001. Then, using the REX toolbox (as integrated into CONN; Duff et al., 2007) we extracted the mean connectivity value within the clusters of this amygdala connectivity network during Fear, Anger, and Happy, and compared differences between these conditions. For all clusters that showed a significant difference between the emotion conditions (Emotion effects), we subsequently investigated connectivity differences between amygdala subregions, separately for Fear, Anger, and Happy (Regions effects). Results were corrected for multiple comparisons at *p*‐FDR < .05.

## 3 RESULTS

### 3.1 Participants

Participants (44 females and 42 males) were between 18 and 49 years of age (Median = 24.32; Mean = 26.12, *SD* = 6.59). There was no significant (*Z* = −1.65, *p* = .10) age difference between males (Median = 26.26; Mean = 27.29, *SD* = 6.54) and females (Median = 23.62; Mean = 25.00, *SD* = 6.53), as revealed by non‐parametric (bootstrapped, 2000 repetitions) regression analysis (implemented due to the age variable and residuals from the regression model not being normally distributed). The sample's mean NART IQ score was 114.84 (*SD* = 6.22), with no difference between males and females identified (*t*(84) = 0.81, *p* = .42; a NART IQ score was missing for one participant).

### 3.2 Enhanced localized activation of superficial and centromedial amygdala to all emotions

**FIGURE 1:**
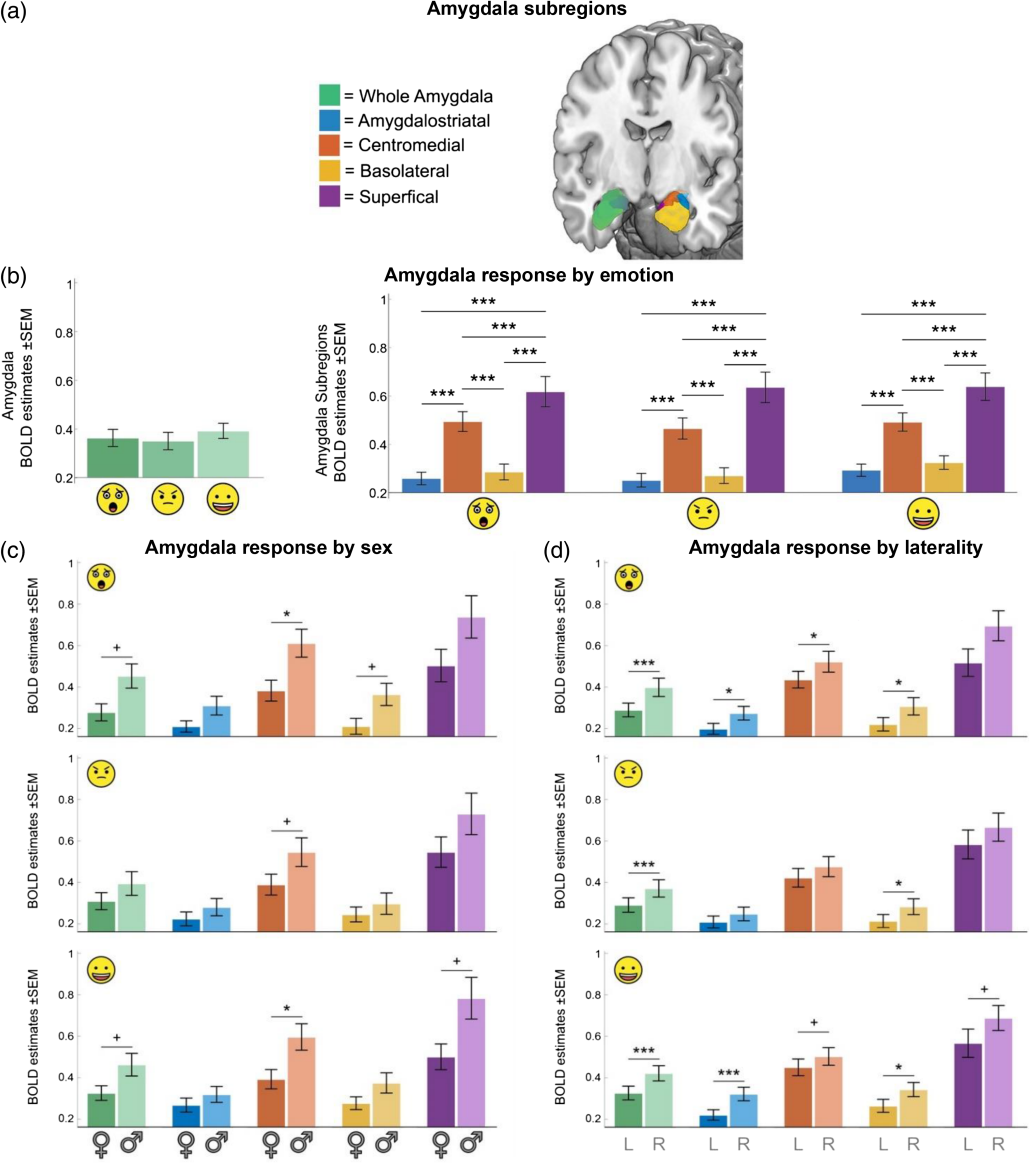
Amygdala and subregions responses during the 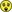 (Fear), 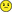 (Anger), and 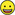 (Happy) conditions (as indicated by the relevant emojis) relative to Shapes. The Emotional Face Matching Task (EFMT) involved pictures of real faces from the Radboud Faces Database. (a) Four anatomically‐defined amygdala subregions were generated for each hemisphere using Anatomy Toolbox (Version 2.1); these were considered together when reporting on whole amygdala activation. (b) Amygdala and all subregions were significantly activated by all emotions (relative to Shapes; *p*‐FDR < .05). However, there were no differences between emotions. The superficial amygdala exhibited significantly higher response to all emotions, compared to all other subregions, and the centromedial amygdala was significantly more highly activated than amygdalostriatal and basolateral subregions (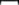 BOLD, blood oxygen level dependent). (c) Greater activation in males (♂, *n* = 42) versus females (♀, *n* = 44) in amygdala and subregions responses during the EFMT across emotions. Statistically significant differences between conditions are indicated (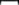). (d) Greater activation in right (R) versus left (L) in amygdala and subregions responses during EFMT across emotions. Statistically significant differences between conditions are indicated (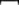). Error bars represent the 95% confidence interval of the mean. Results are significant at ***p‐FDR < .001; *p‐FDR < .05; ^+^ *p*‐FDR < .06.

We first investigated whether the whole amygdala and its subregions were significantly activated in response to emotional faces, and evaluated the differential responses across the four amygdala subregions (basolateral, centromedial, superficial, and amygdalostriatal transition area; Figure 1a) to emotional stimuli (fearful, angry, and happy facial expressions). The results show that the whole amygdala and each of its subregions were significantly activated to all emotional stimuli (relative to Shapes; all *p*‐FDR < .05; Figure 1b and Supplementary Tables 1.1 to 1.5). Importantly, however, we found no statistically significant differences between the emotions in the level of activation in the whole amygdala, or any of its subregions.

We next examined the pattern of responses across the subregions to the different emotions. Our findings suggested a substantially greater level of activation in the superficial amygdala relative to the other subregions. We also found that the centromedial amygdala was more strongly activated than the amygdalostriatal and basolateral subregions across all emotions (*p*‐FDR < .001; Figure 1b and Supplementary Table 1.6) extending earlier findings (Goossens et al., 2009; Hurlemann et al., 2008). This indicates that when viewing faces of negative and positive emotions (fear, anger, and happiness), superficial and centromedial amygdala activation is significantly greater than that of the amygdalostriatal or basolateral amygdala subregions.

Compared to females, males exhibited significantly greater activation to emotions combined (vs. shapes) for the whole amygdala (*p*‐FDR = 0.01), the centromedial (*p*‐FDR = 0.008), basolateral (*p*‐FDR = 0.035) and superficial subregions (*p*‐FDR = 0.021; Figure 1c; Supplementary Tables 1.1 to 1.5). However, when investigating sex effects across each emotion separately, it was statistically significant only for the centromedial subregion during the Fear and Happy conditions (*p*‐FDR < .03). These findings highlight the importance of considering subregional amygdala responses during emotion processing and related sex differences.

A laterality effect was observed in the whole amygdala (*p*‐FDR < .001) and across all subregions (amygdalostriatal, *p*‐FDR = 0.001, centromedial, *p*‐FDR = 0.003, basolateral, *p*‐FDR = 0.001 and superficial, *p*‐FDR = 0.002), with greater activation in the right (vs. left) hemisphere (Figure 1d; Supplementary Tables 1.1 to 1.5). This difference was present for every individual emotion for the whole amygdala (*p*‐FDR = 0.001) and in the basolateral amygdala (*p*‐FDR ≤0.015), in the Fear and Happy conditions for the amygdalostriatal subregion (*p*‐FDR ≤0.012) and superficial amygdala (*p*‐FDR ≤0.035), and in the Fear condition for the centromedial subregion (*p*‐FDR = 0.034).

### 3.3 Enhanced connectivity of the basolateral amygdala to wider brain networks in response to fear

We next focused on whether amygdala subregions differ in their intrinsic connectivity with the rest of the brain (Seeley et al., 2007). To determine subregional connectivity alterations in healthy adults during an emotion‐processing task and to examine differences in connectivity patterns in response to specific emotions, we used gPPI connectivity analyses for the whole amygdala, and then for the individual amygdala subregions, separately.

**FIGURE 2:**
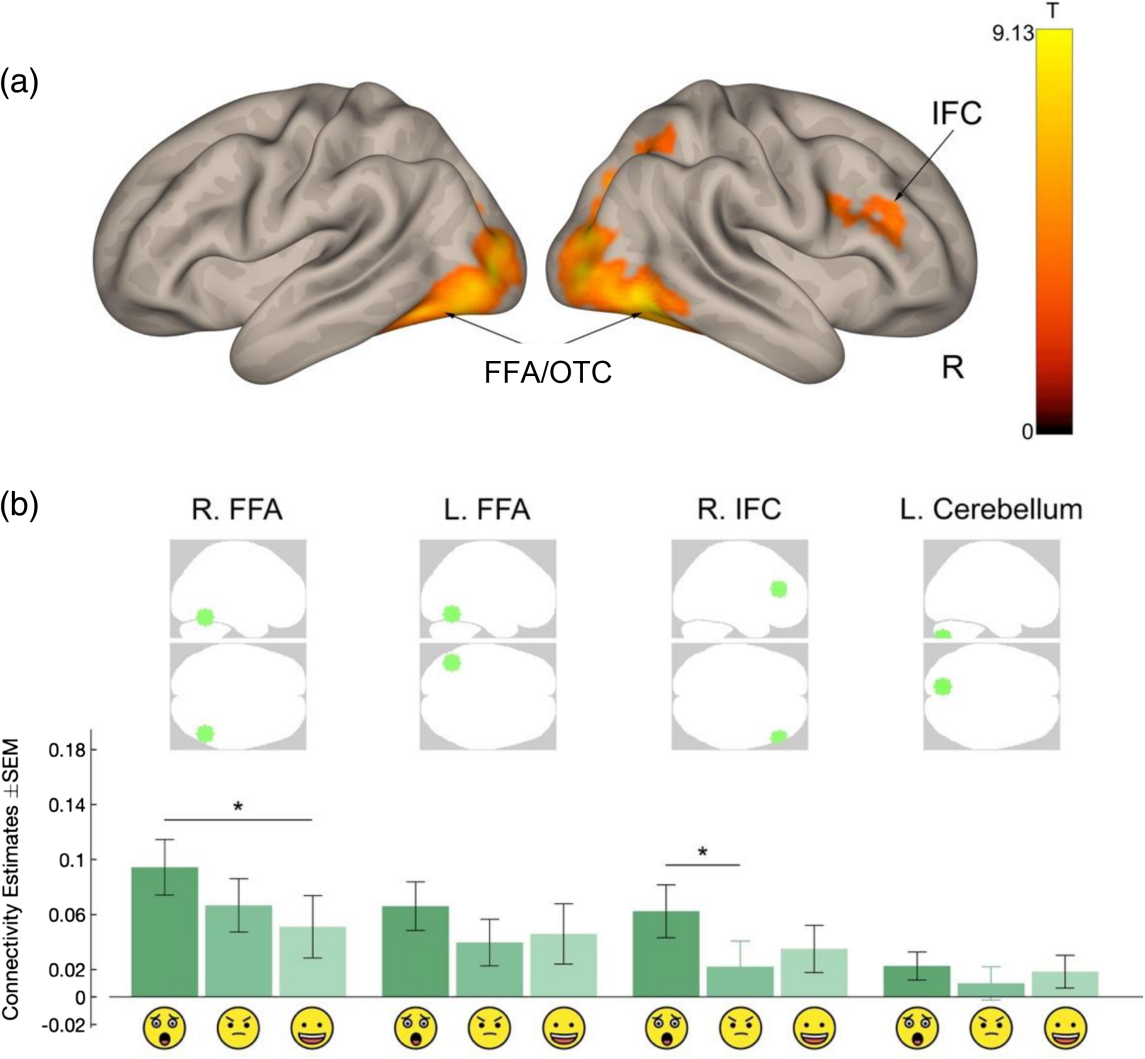
Whole amygdala connectivity during emotional face recognition (vs. Shapes) as revealed by generalized psycho‐physiological interaction (gPPI) analyses. (a) Whole‐brain voxelwise analysis: amygdala connectivity network in response to emotional faces (cluster forming threshold, *p* < .001; cluster‐corrected threshold, *p*‐FEW < .05). The color bar represents the *T*‐score. (b) Region of interest (ROI) analysis: Bar graphs depict level of connectivity of the amygdala with a selected ROI by Emotion (statistically significant at *p*‐FDR < .05, unless the error bar is the same color as the bar graph). Emotion‐related connectivity differences in amygdala connectivity with selected ROIs are indicated by * and — over the relevant emotions. FFA, fusiform face area; IFC, inferior frontal cortex; OTC, occipitotemporal cortex; R, right; 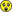, Fear; 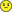, Anger; 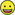, Happy; *, statistically significant differences between emotions at *p*‐FDR < .05.

We found that, in response to emotional faces combined (vs. Shapes), the whole amygdala displayed greater functional connectivity with a network comprising bilateral occipitotemporal and parietal cortices, right lateral prefrontal cortex, and left cerebellum (Figure 2a). The occipitotemporal and parietal areas spread ventrally from the inferior lateral occipital cortex, through to the occipitotemporal fusiform gyrus and the inferior temporal cortex, bilaterally. The implicated areas also spread dorsally, into the middle occipital gyrus, bilaterally, and further into the angular gyrus of the parietal cortex, in the right hemisphere only. Importantly, the area with peak connectivity with the amygdala in this posterior component of the network (in fact, the peak connectivity across all network components) was the right fusiform face area (FFA). The lateral prefrontal region exhibiting connectivity with the whole amygdala comprised pars opercularis and pars triangularis of the inferior frontal cortex (IFC, with peak connectivity over pars triangularis); see Supplementary Table 2.1.

We next sought to test whether amygdala connectivity with these networks was differentially modulated by any of the emotions. For this, we generated spherical regions of interest (ROIs) of 10 mm radius at selected locations within the network. We chose the peak voxels in the right FFA and IFC, and the left cerebellum. For the location of the ROI in the left occipitotemporal cluster, we selected a site contralateral to the right FFA ROI (at MNI ‐48 ‐56 ‐16), given the importance of the FFA, bilaterally, in face processing in general, and emotional face processing in particular (Chan & Downing, 2011; Zhen et al., 2013) and studies using the same emotional task as here (Borod et al., 1998; Bzdok et al., 2013; Davidson & Hugdahl, 1996). We observed a pattern of greater amygdala connectivity during Fear compared to other emotions across ROIs, which was statistically significant for amygdala‐right FFA connectivity compared to Happy, and for amygdala‐right IFC connectivity compared to Anger (*p*‐FDR < .05; Figure 2b; Supplementary Table 2.1). No Sex or Laterality effects were observed. Thus, while the whole amygdala appears to show a similar connectivity profile with posterior (occipitotemporal area) and anterior (IFC) brain regions, the connectivity between these regions is greatest in response to fearful faces compared with angry and happy faces.

**FIGURE 3:**
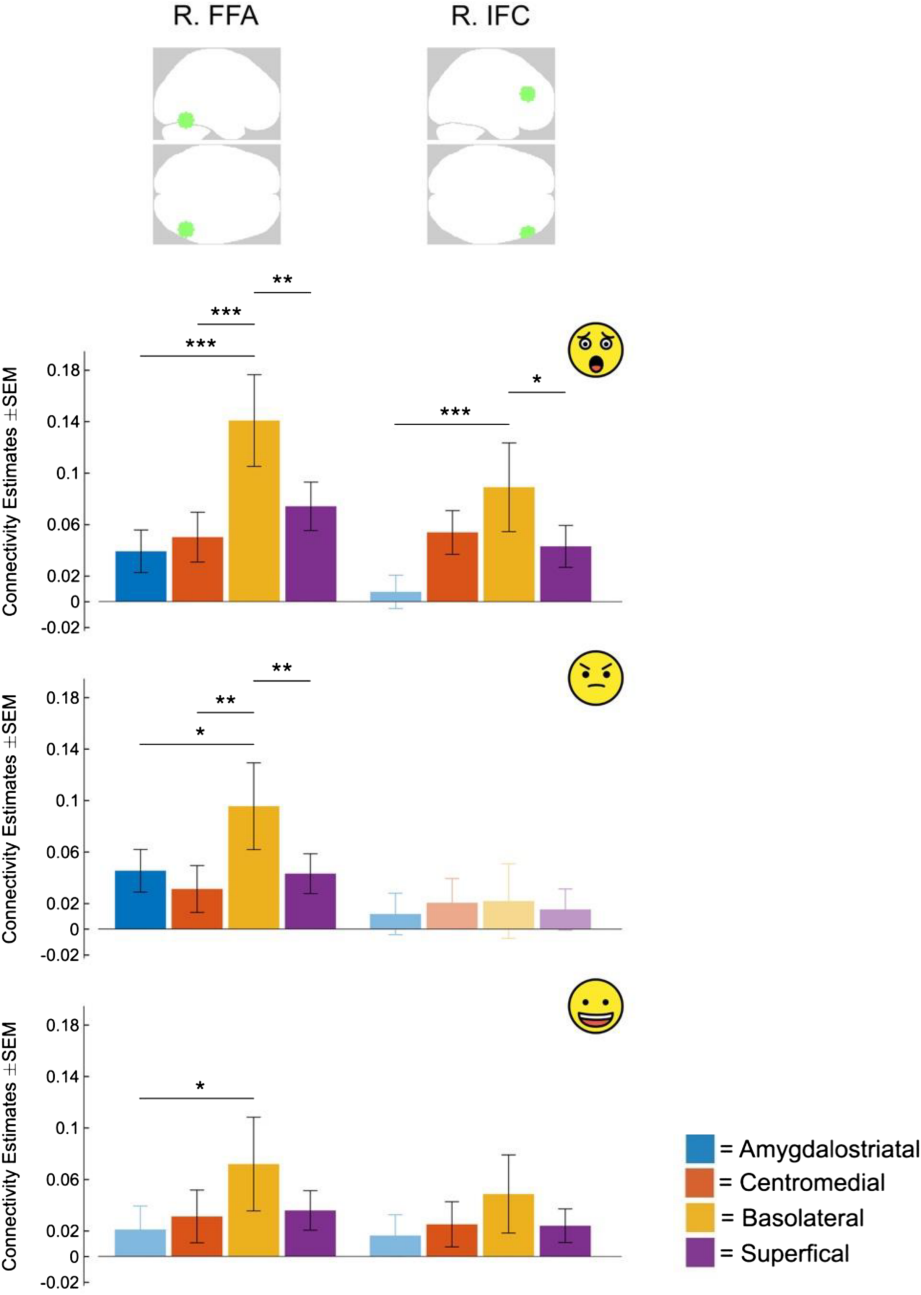
Amygdala subregions connectivity during emotional face recognition (vs. shapes) as revealed by generalized psycho‐physiological interaction (gPPI) analysis. Bar graphs depict level of connectivity of each subregion with a selected region of interest (ROI) by Emotion (statistically significant at *p*‐FDR < .05, unless the error bar is the same color as the bar graph). Connectivity differences between amygdala subregions is indicated by * and — over the relevant regions. FFA, fusiform face area; IFC, inferior frontal cortex; R, right; 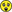, Fear; 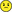, Anger; 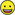, Happy; ***p‐FDR < .001, **p‐FDR < .01, *p‐FDR < .05.

Next, we sought to investigate if amygdala connectivity with the right FFA and IFC differed for each of the amygdala *subregions* across the three emotions. During Fear, all subregions exhibited significant connectivity to both right FFA and IFC (except for the amygdalostriatal subregion, which did not exhibit significant connectivity with right IFC) (*p*‐FDR < .05; Figure 3; Supplementary Table 2.2). During the Anger condition, all amygdala subregions displayed connectivity to the right FFA only. During Happy, except for the amygdalostriatal subregion, all subregions exhibited functional connectivity to both the right FFA and IFC.

Examination of the level of connectivity in each subregion suggested a pattern of greater connectivity of the basolateral amygdala compared to other subregions, so we explicitly tested for this difference. During the Fear condition, we found basolateral amygdala connectivity with right FFA was significantly greater than all other subregions (*p*‐FDR < .05; Figure 3; Supplementary Table 2.3). In addition, significantly increased connectivity of the basolateral subregion with right IFC was observed during the Fear condition relative to the other subregions, but only the differences with amygdalostriatal and superficial subregions survived correction for multiple comparisons (*p*‐FDR < .05). During Anger, basolateral connectivity with right FFA (but not right IFC) was increased relative to the other regions (*p*‐FDR < .05). During Happy, basolateral connectivity with right FFA (but not right IFC) was increased relative to the amygdalostriatal region only (*p*‐FDR < .05). Together, this pattern of findings suggests that of the amygdala subregions, the basolateral amygdala appears to have the greatest functional connectivity with anterior and posterior brain regions during the processing of emotional faces and that this network is particularly sensitive to fearful faces.

## 4 DISCUSSION

A growing body of evidence suggests that a departure from the traditional view that the amygdala exclusively specializes in the processing of fearful stimuli may be warranted. Yet the current findings suggest a more complex picture exists, as we found that the specificity of the amygdala's subregional response to fear is reflected not in its localized activity, but in its connectivity with posterior (bilateral occipitotemporal/FFA) and anterior (right IFC) brain regions that are involved in the processing of facial information (Chan & Downing, 2011; Zhen et al., 2013). With regards to fMRI activity, we show that there is segregation between amygdala subregions. Specifically, we localized the most robust activation to the superficial subregion across all emotions, relative to other amygdala subregions, in line with previous evidence (Bzdok et al., 2013; Goossens et al., 2009; Hurlemann et al., 2008). We also found the centromedial amygdala to be significantly more activated across all emotions compared to the amygdalostriatal and basolateral subregions. However, it was significantly less activated compared to the superficial subregion. With regards to connectivity, we show that it is the basolateral amygdala (more so than the other subregions) that oversees long‐distance communication with extended components of a brain network for face processing, a link that is particularly sensitive to the recognition of fear. The predominant activation of the superficial subregion and heightened connectivity of the basolateral subregion underscore their distinct contributions to emotion processing. This aligns with established literature that shows that the basolateral subregion is primarily involved in receiving external inputs and connectivity with the visual cortex, particularly in the perception of facial expressions (Bzdok et al., 2013). Simultaneously, the heightened responsiveness of the superficial subregion aligns with its established function as an area involved in being attuned to and processing visual displays of facial emotional expressions (Goossens et al., 2009; Hurlemann et al., 2008). Exploratory analyses provide further evidence of specific sex differences in the superficial amygdala response (males > females to happy faces), along with greater right (vs. left) hemisphere response across all amygdala subregions (to all emotions) that was only evident during the localized responses and not in the communication with the identified large‐scale brain networks.

We found that all amygdala subregions significantly responded to all emotional stimuli with no differences in activation between the emotions (Fear, Anger, and Happiness), supporting the view that the amygdala is less specialized to fear. However, our findings of a significantly greater superficial amygdala response, compared to the other subregions, to all emotions support the role of the superficial amygdala as a hub that is highly tuned to evaluate the social relevance and reward of incoming emotional stimuli in healthy humans (Bzdok et al., 2013; Goossens et al., 2009; Hurlemann et al., 2008). We also found that the centromedial amygdala was significantly more activated than the amygdalostriatal and basolateral amygdala, but significantly less activated than the superficial amygdala. This finding suggests that the centromedial amygdala constitutes the main output center for the appropriate behavioral response (Derntl et al., 2008). Taken together, these findings provide novel evidence of differential amygdala subregion responses to emotional face stimuli, with the results suggesting subregions important for social relevance processing (superficial) and for sending signals out to initiate appropriate behavioral responses (centromedial) are more highly activated, compared to subregions that are important for stimulating fear response (basolateral) and for processing positive valence (amygdalostriatal).

Evidence of subregion‐specific and emotion‐specific sex differences also shed new light on the inconsistencies in prior reports on sex differences in amygdala response to emotional stimuli (Killgore & Yurgelun‐Todd, 2001; Sergerie et al., 2008). The finding that across all subregions both hemispheres significantly activate to all emotional stimuli, but that this is more robust in the right (vs. left) hemisphere, provides evidence to challenge traditional theories of asymmetry in the brain during the processing of emotional stimuli, such as the right hemisphere hypothesis and valence hypothesis (Borod et al., 1998; Davidson & Hugdahl, 1996; Demaree et al., 2005). Interestingly though, the localized differences in hemispheric activity and evidence of sex differences did not play out in the subsequent large‐scale network‐based (connectivity) analyses.

Our connectivity data showed convergent processing between the centromedial, basolateral, and superficial amygdala subregions and the bilateral occipitotemporal area and right IFC (pars opercularis, BA 44). Connectivity between the amygdala subregions and the identified large‐scale network regions is consistent with prior task and resting‐state fMRI paradigms (Bach et al., 2011; Faria et al., 2012; Ghashghaei et al., 2007; Kerestes et al., 2017; Seeley et al., 2007; Truitt et al., 2007). However, none of the studies were able to examine emotion‐specific effects (Kerestes et al., 2017). We were able to differentiate possible emotion‐specific effects in amygdala subregions. We showed that the basolateral amygdala plays an important role in the higher‐order integration of emotional face stimuli via interactions with multiple cortical and subcortical brain structures, which was more pronounced during the processing of fear. This flow of emotional information between the amygdala and cortical areas is supported by anatomical connections of the amygdala subregions shown in previous tractography‐based parcellations of the human and animal amygdala (Bach et al., 2011; Bracht et al., 2009; Ghashghaei et al., 2007). The greater connectivity of the basolateral amygdala to fear aligns with the view that the amygdala is sensitive to emotions (Borod et al., 1998). However, we add that this sensitivity to different emotions is only evident during the communication with distant cortical regions. This is crucially important for our understanding of the mechanisms underlying our appropriate and adaptive behavioral responses to environmental cues, a function driving human evolution (Janak & Tye, 2015).

There are several limitations of this study. First, our use of the Anatomy Toolbox to segment the amygdala and its subregions may lead to differences in findings compared to studies that used other atlases (Saygin et al., 2017; Tzourio‐Mazoyer et al., 2002). Future studies should interpret findings with this consideration in mind. Our analysis was restricted to the amygdala and its subregions, however future studies should also explore the bed nucleus of the stria terminalis (part of the central extended amygdala) given preliminary evidence of its role in emotion processing (Fox & Shackman, 2019; Sladky et al., 2018).

To conclude, we provide a detailed study of emotion processing in the human amygdala. The functionally heterogeneous findings of the amygdala subregions in healthy humans from this study now highlight the value of future investigations of amygdala subregion function and their connectivity in a range of neurologic and psychiatric disorders. In many of the clinical groups known to be associated with amygdala dysfunction, this approach could assist with the refinement of targets for anxiolytic treatments (Faria et al., 2012). For instance, the basolateral and basomedial amygdala have been shown to have decreased activation following antidepressant treatment in patients with social anxiety disorder (Demaree et al., 2005). Here we provide a foundation of the functional underpinnings of these findings in healthy humans. It will be critical to advance the understanding of habituation effects (Plichta et al., 2014) at the amygdala subregional level as the superficial amygdala appears to drive most of the localized activity during the processing of emotional faces. Critically, the current findings open up a new and exciting dimension to the role of amygdala in emotion processing (Le Doux, 2012), ranging from models of validated amygdala connectivity networks (Stein et al., 2007) to the impact of genetic variants (e.g., serotonin transporter, and vasopressin receptor) and personality traits (Meyer‐Lindenberg et al., 2009; Pezawas et al., 2005) on amygdala function. These future directions will be relevant to the understanding of common mental health disorders such as social anxiety, autism, and depression.

## CONFLICT OF INTEREST STATEMENT

The authors declare no conflicts of interest.

## Supporting information


**Data S1.** Supporting Information.

## Data Availability

The data that support the findings of this study are available from the corresponding author (Simone Mizzi) upon reasonable request.

## References

[hbm26673-bib-0001] Adolphs, R. (2008). Fear, faces, and the human amygdala. Current Opinion in Neurobiology, 18(2), 166–172. 10.1016/j.conb.2008.06.006 18655833 PMC2580742

[hbm26673-bib-0002] Andersson, J. L. , Skare, S. , & Ashburner, J. (2003). How to correct susceptibility distortions in spin‐echo echo‐planar images: Application to diffusion tensor imaging. NeuroImage, 20(2), 870–888. 10.1016/S1053-8119(03)00336-7 14568458

[hbm26673-bib-0003] Ashburner, J. (2007). A fast diffeomorphic image registration algorithm. NeuroImage, 38(1), 95–113. 10.1016/j.neuroimage.2007.07.007 17761438

[hbm26673-bib-0004] Bach, D. R. , Behrens, T. E. , Garrido, L. , Weiskopf, N. , & Dolan, R. J. (2011). Deep and superficial amygdala nuclei projections revealed in vivo by probabilistic tractography. The Journal of Neuroscience, 31(2), 618–623. 10.1523/jneurosci.2744-10.2011 21228170 PMC3059574

[hbm26673-bib-0005] Behzadi, Y. , Restom, K. , Liau, J. , & Liu, T. T. (2007). A component based noise correction method (CompCor) for BOLD and perfusion based fMRI. NeuroImage, 37(1), 90–101. 10.1016/j.neuroimage.2007.04.042 17560126 PMC2214855

[hbm26673-bib-0006] Borod, J. C. , Koff, E. , Yecker, S. , Santschi, C. , & Schmidt, J. M. (1998). Facial asymmetry during emotional expression: Gender, valence, and measurement technique. Neuropsychologia, 36(11), 1209–1215. 10.1016/S0028-3932(97)00166-8 9842766

[hbm26673-bib-0007] Bracht, T. , Tüscher, O. , Schnell, S. , Kreher, B. , Rüsch, N. , Glauche, V. , Lieb, K., Ebert, D., Il'yasov, K. A., Hennig, J., Weiller, C., van Elst, L. T., & Saur, D. (2009). Extraction of prefronto‐amygdalar pathways by combining probability maps. Psychiatry Research: Neuroimaging, 174(3), 217–222. 10.1016/j.pscychresns.2009.05.001 19910167

[hbm26673-bib-0008] Brett, M. , Anton, J.L. , Valabregue, R. , & Poline, J.‐B. (2006). Region of interest analysis using an SPM toolbox. The 8th International Conference on Functional Mapping of the Human Brain, June 2–6, 2002, Sendai, Japan.

[hbm26673-bib-0009] Bzdok, D. , Laird, A. R. , Zilles, K. , Fox, P. T. , & Eickhoff, S. B. (2013). An investigation of the structural, connectional, and functional subspecialization in the human amygdala. Human Brain Mapping, 34(12), 3247–3266. 10.1002/hbm.22138 22806915 PMC4801486

[hbm26673-bib-0010] Chan, A. W. , & Downing, P. (2011). Faces and eyes in human lateral prefrontal cortex. Frontiers in Human Neuroscience, 5, 51. 10.3389/fnhum.2011.00051 21687796 PMC3108382

[hbm26673-bib-0011] Davidson, R. J. , & Hugdahl, K. (1996). Brain asymmetry. MIT Press.

[hbm26673-bib-0012] Demaree, H. A. , Everhart, D. E. , Youngstrom, E. A. , & Harrison, D. W. (2005). Brain lateralization of emotional processing: Historical roots and a future incorporating “dominance”. Behavioral and Cognitive Neuroscience Reviews, 4(1), 3–20. 10.1177/1534582305276837 15886400

[hbm26673-bib-0013] Derntl, B. , Windischberger, C. , Robinson, S. , Lamplmayr, E. , Kryspin‐Exner, I. , Gur, R. C. , Moser, E., & Habel, U. (2008). Facial emotion recognition and amygdala activation are associated with menstrual cycle phase. Psychoneuroendocrinology, 33(8), 1031–1040. 10.1016/j.psyneuen.2008.04.014 18675521 PMC7437605

[hbm26673-bib-0014] Diano, M. , Tamietto, M. , Celeghin, A. , Weiskrantz, L. , Tatu, M. K. , Bagnis, A. , Duca, S., Geminiani, G., Cauda, F., & Costa, T. (2017). Dynamic changes in amygdala psychophysiological connectivity reveal distinct neural networks for facial expressions of basic emotions. Scientific Reports, 7, 45260. 10.1038/srep45260 28345642 PMC5366904

[hbm26673-bib-0015] Duff, E. P. , Cunnington, R. , & Egan, G. F. (2007). REX: Response exploration for neuroimaging datasets. Neuroinformatics, 5(4), 223–234. 10.1007/s12021-007-9001-y 17985253

[hbm26673-bib-0016] Faria, V. , Appel, L. , Åhs, F. , Linnman, C. , Pissiota, A. , Frans, Ö. , Bani, M., Bettica, P., Pich, E. M., Jacobsson, E., Wahlstedt, K., Fredrikson, M., & Furmark, T. (2012). Amygdala subregions tied to SSRI and placebo response in patients with social anxiety disorder. Neuropsychopharmacology, 37(10), 2222–2232. 10.1038/npp.2012.72 22617357 PMC3422487

[hbm26673-bib-0017] Fox, A. S. , & Shackman, A. J. (2019). The central extended amygdala in fear and anxiety: Closing the gap between mechanistic and neuroimaging research. Neuroscience Letters, 693, 58–67. 10.1016/j.neulet.2017.11.056 29195911 PMC5976525

[hbm26673-bib-0018] Fusar‐Poli, P. , Placentino, A. , Carletti, F. , Allen, P. , Landi, P. , Abbamonte, M. , Barale, F., Perez, J., McGuire, P., & Politi, P. L. (2009). Laterality effect on emotional faces processing: ALE meta‐analysis of evidence. Neuroscience Letters, 452(3), 262–267. 10.1016/j.neulet.2009.01.065 19348735

[hbm26673-bib-0019] Ghashghaei, H. T. , Hilgetag, C. C. , & Barbas, H. (2007). Sequence of information processing for emotions based on the anatomic dialogue between prefrontal cortex and amygdala. NeuroImage, 34(3), 905–923. 10.1016/j.neuroimage.2006.09.046 17126037 PMC2045074

[hbm26673-bib-0020] Gilpin, N. W. , Herman, M. A. , & Roberto, M. (2015). The central amygdala as an integrative hub for anxiety and alcohol use disorders. Biological Psychiatry, 77(10), 859–869. 10.1016/j.biopsych.2014.09.008 25433901 PMC4398579

[hbm26673-bib-0021] Goossens, L. , Kukolja, J. , Onur, O. A. , Fink, G. R. , Maier, W. , Griez, E. , Schruers, K., & Hurlemann, R. (2009). Selective processing of social stimuli in the superficial amygdala. Human Brain Mapping, 30(10), 3332–3338. 10.1002/hbm.20755 19347877 PMC6870612

[hbm26673-bib-0022] Hariri, A. R. , Tessitore, A. , Mattay, V. S. , Fera, F. , & Weinberger, D. R. (2002). The amygdala response to emotional stimuli: A comparison of faces and scenes. NeuroImage, 17(1), 317–323. 10.1006/nimg.2002.1179 12482086

[hbm26673-bib-0023] Herrington, J. D. , Miller, J. S. , Pandey, J. , & Schultz, R. T. (2016). Anxiety and social deficits have distinct relationships with amygdala function in autism spectrum disorder. Social Cognitive and Affective Neuroscience, 11(6), 907–914. 10.1093/scan/nsw015 26865425 PMC4884314

[hbm26673-bib-0050] Hrybouski, S. , Aghamohammadi‐Sereshki, A. , Madan, C. R. , Shafer, A. T. , Baron, C. A. , Seres, P. , Beaulieu, C. , Olsen, F. , & Malykhin, N. V. (2016). Amygdala subnuclei response and connectivity during emotional processing. NeuroImage, 133, 98–110. 10.1016/j.neuroimage.2016.02.056 26926791

[hbm26673-bib-0024] Hurlemann, R. , Rehme, A. K. , Diessel, M. , Kukolja, J. , Maier, W. , Walter, H. , & Cohen, M. X. (2008). Segregating intra‐amygdalar responses to dynamic facial emotion with cytoarchitectonic maximum probability maps. Journal of Neuroscience Methods, 172(1), 13–20. 10.1016/j.jneumeth.2008.04.004 18486975

[hbm26673-bib-0025] Janak, P. H. , & Tye, K. M. (2015). From circuits to behaviour in the amygdala. Nature, 517, 284–292. 10.1038/nature14188 25592533 PMC4565157

[hbm26673-bib-0026] Jenkinson, M. , Beckmann, C. F. , Behrens, T. E. , Woolrich, M. W. , & Smith, S. M. (2012). Fsl. Neuroimage, 62(2), 782–790. 10.1016/j.neuroimage.2011.09.015 21979382

[hbm26673-bib-0027] Jenkinson, M. , Wilson, J. L. , & Jezzard, P. (2004). Perturbation method for magnetic field calculations of nonconductive objects. Magnetic Resonance in Medicine, 52(3), 471–477. 10.1002/mrm.20194 15334564

[hbm26673-bib-0028] Kerestes, R. , Chase, H. W. , Phillips, M. L. , Ladouceur, C. D. , & Eickhoff, S. B. (2017). Multimodal evaluation of the amygdala's functional connectivity. NeuroImage, 148, 219–229. 10.1016/j.neuroimage.2016.12.023 28089676 PMC5416470

[hbm26673-bib-0029] Killgore, W. D. S. , & Yurgelun‐Todd, D. A. (2001). Sex differences in amygdala activation during the perception of facial affect. Neuroreport, 12(11), 2543–2547. 10.1097/00001756-200108080-00050 11496145

[hbm26673-bib-0030] Labuschagne, I. , Phan, K. L. , Wood, A. , Angstadt, M. , Chua, P. , Heinrichs, M. , Stout, J. C., & Nathan, P. J. (2010). Oxytocin attenuates amygdala reactivity to fear in generalized social anxiety disorder. Neuropsychopharmacology, 35(12), 2403–2413. 10.1038/npp.2010.123 20720535 PMC3055328

[hbm26673-bib-0031] Le Doux, J. (2003). The emotional brain, fear, and the amygdala. Cellular and Molecular Neurobiology, 23(4), 727–738. 10.1023/A:1025048802629 14514027 PMC11530156

[hbm26673-bib-0032] Le Doux, J. (2012). Rethinking the emotional brain. Neuron, 73(4), 653–676. 10.1016/j.neuron.2012.02.004 22365542 PMC3625946

[hbm26673-bib-0033] Meyer‐Lindenberg, A. , Kolachana, B. , Gold, B. , Olsh, A. , Nicodemus, K. K. , Mattay, V. , Dean, M., & Weinberger, D. R. (2009). Genetic variants in AVPR1A linked to autism predict amygdala activation and personality traits in healthy humans. Molecular Psychiatry, 14(10), 968–975. 10.1038/mp.2008.54 18490926 PMC2754603

[hbm26673-bib-0034] Pezawas, L. , Meyer‐Lindenberg, A. , Drabant, E. M. , Verchinski, B. A. , Munoz, K. E. , Kolachana, B. S. , Egan, M. F., Mattay, V. S., Hariri, A. R., & Weinberger, D. R. (2005). 5‐HTTLPR polymorphism impacts human cingulate‐amygdala interactions: A genetic susceptibility mechanism for depression. Nature Neuroscience, 8(6), 828–834. 10.1038/nn1463 15880108

[hbm26673-bib-0035] Phelps, E. A. , & LeDoux, J. E. (2005). Contributions of the amygdala to emotion processing: From animal models to human behavior. Neuron, 48(2), 175–187. 10.1016/j.neuron.2005.09.025 16242399

[hbm26673-bib-0036] Plichta, M. M. , Grimm, O. , Morgen, K. , Mier, D. , Sauer, C. , Haddad, L. , Tost, H., Esslinger, C., Kirsch, P., Schwarz, A. J., & Meyer‐Lindenberg, A. (2014). Amygdala habituation: A reliable fMRI phenotype. NeuroImage, 103, 383–390. 10.1016/j.neuroimage.2014.09.059 25284303

[hbm26673-bib-0037] Qin, S. , Young, C. B. , Duan, X. , Chen, T. , Supekar, K. , & Menon, V. (2014). Amygdala subregional structure and intrinsic functional connectivity predicts individual differences in anxiety during early childhood. Biological Psychiatry, 75(11), 892–900. 10.1016/j.biopsych.2013.10.006 24268662 PMC3984386

[hbm26673-bib-0038] Saygin, Z. M. , Kliemann, D. , Iglesias, J. E. , van der Kouwe, A. J. W. , Boyd, E. , Reuter, M. , Stevens, A., Van Leemput, K., McKee, A., Frosch, M. P., Fischl, B., & Augustinack, J. C. (2017). High‐resolution magnetic resonance imaging reveals nuclei of the human amygdala: Manual segmentation to automatic atlas. NeuroImage, 155, 370–382. 10.1016/j.neuroimage.2017.04.046 28479476 PMC5557007

[hbm26673-bib-0039] Seeley, W. W. , Menon, V. , Schatzberg, A. F. , Keller, J. , Glover, G. H. , Kenna, H. , Reiss, A. L., & Greicius, M. D. (2007). Dissociable intrinsic connectivity networks for salience processing and executive control. The Journal of Neuroscience, 27(9), 2349–2356. 10.1523/jneurosci.5587-06.2007 17329432 PMC2680293

[hbm26673-bib-0040] Sergerie, K. , Chochol, C. , & Armony, J. L. (2008). The role of the amygdala in emotional processing: A quantitative meta‐analysis of functional neuroimaging studies. Neuroscience & Biobehavioral Reviews, 32(4), 811–830. 10.1016/j.neubiorev.2007.12.002 18316124

[hbm26673-bib-0041] Sladky, R. , Geissberger, N. , Pfabigan, D. M. , Kraus, C. , Tik, M. , Woletz, M. , Paul, K., Vanicek, T., Auer, B., Kranz, G. S., Lamm, C., Lanzenberger, R., & Windischberger, C. (2018). Unsmoothed functional MRI of the human amygdala and bed nucleus of the stria terminalis during processing of emotional faces. NeuroImage, 168, 383–391. 10.1016/j.neuroimage.2016.12.024 28108394

[hbm26673-bib-0042] Stein, J. L. , Wiedholz, L. M. , Bassett, D. S. , Weinberger, D. R. , Zink, C. F. , Mattay, V. S. , & Meyer‐Lindenberg, A. (2007). A validated network of effective amygdala connectivity. NeuroImage, 36(3), 736–745. 10.1016/j.neuroimage.2007.03.022 17475514

[hbm26673-bib-0043] Stevens, J. S. , & Hamann, S. (2012). Sex differences in brain activation to emotional stimuli: A meta‐analysis of neuroimaging studies. Neuropsychologia, 50(7), 1578–1593. 10.1016/j.neuropsychologia.2012.03.011 22450197

[hbm26673-bib-0044] Tessitore, A. , Hariri, A. R. , Fera, F. , Smith, W. G. , Chase, T. N. , Hyde, T. M. , Weinberger, D. R., & Mattay, V. S. (2002). Dopamine modulates the response of the human amygdala: A study in Parkinson's disease. The Journal of Neuroscience, 22(20), 9099–9103. 10.1523/jneurosci.22-20-09099.2002 12388617 PMC6757686

[hbm26673-bib-0045] Tian, T. , Young, C. B. , Zhu, Y. , Xu, J. , He, Y. , Chen, M. , Hao, L., Jiang, M., Qiu, J., Chen, X., & Qin, S. (2021). Socioeconomic disparities affect children's amygdala‐prefrontal circuitry via stress hormone response. Biological Psychiatry, 90(3), 173–181. 10.1016/j.biopsych.2021.02.002 33832707

[hbm26673-bib-0046] Truitt, W. A. , Sajdyk, T. J. , Dietrich, A. D. , Oberlin, B. , McDougle, C. J. , & Shekhar, A. (2007). From anxiety to autism: Spectrum of abnormal social behaviors modeled by progressive disruption of inhibitory neuronal function in the basolateral amygdala in Wistar rats. Psychopharmacology, 191(1), 107–118. 10.1007/s00213-006-0674-y 17277936

[hbm26673-bib-0047] Tzourio‐Mazoyer, N. , Landeau, B. , Papathanassiou, D. , Crivello, F. , Etard, O. , Delcroix, N. , Mazoyer, B., & Joliot, M. (2002). Automated anatomical labeling of activations in SPM using a macroscopic anatomical parcellation of the MNI MRI single‐subject brain. NeuroImage, 15(1), 273–289. 10.1006/nimg.2001.0978 11771995

[hbm26673-bib-0048] Whitfield‐Gabrieli, S. , & Nieto‐Castanon, A. (2012). Conn: A functional connectivity toolbox for correlated and anticorrelated brain networks. Brain Connectivity, 2(3), 125–141. 10.1089/brain.2012.0073 22642651

[hbm26673-bib-0049] Zhen, Z. , Fang, H. , & Liu, J. (2013). The hierarchical brain network for face recognition. PLoS One, 8(3), e59886. 10.1371/journal.pone.0059886 23527282 PMC3603994

