# Supplementary material for: Specialization of amygdala subregions in emotion processing
Source: Hum Brain Mapp. 2024 Apr 9;45(5):e26673. doi: 10.1002/hbm.26673 (PMC11002533; doi:10.1002/hbm.26673)
Supplement: Supplementary file 1 — Data S1. Supporting Information. [file HBM-45-e26673-s001.pdf]

Supplementary Materials for

Specialization of amygdala subregions in emotion processing

Izelle Labuschagne[1,2], Juan F. Dominguez[3], Sally Grace[1], Simone Mizzi[4*], Julie D. Henry[2], Craig Peters[5], Christine A. Rabinak[5], Erin Sinclair[1], Valentina Lorenzetti[1], Gill Terrett[1], Peter G. Rendell[1], Mangor Pedersen[6,7], Darren R. Hocking8, Markus Heinrichs[9,10]

*Corresponding author. Email: simone.mizzi2@rmit.edu.au

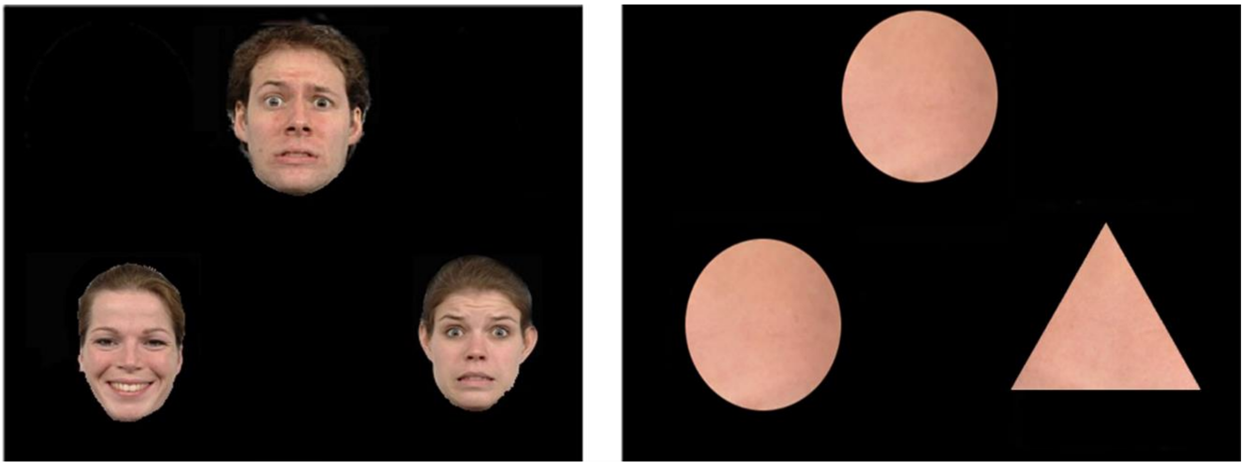

Fig. S1. Face and Shape Stimuli used in the Emotional Face Matching Task.

For each trial, participants were instructed to select one of two faces on the bottom of the display that expressed the same emotion as the target face at the top. Emotions expressed by the faces included fear, anger and happiness. Face stimuli were selected from the Radboud Faces Database (Langner et al., 2010). To control for perceptual, motor and cognitive demands not specifically associated with emotion processing, participants were also required to perform a shape-matching task. In this task, participants had to match one of two shapes on the bottom (circle, triangle, and rectangle) to the one on top. Face-matching trials were arranged into blocks and were interspersed between blocks of shape-matching trials.

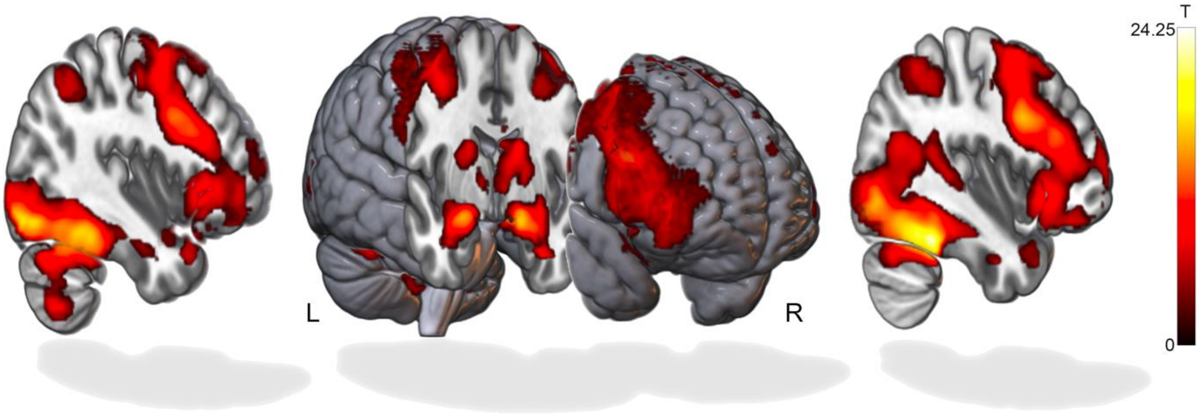

Fig. S2. Regions activated by Faces (vs. Shapes) contrast.

There was significantly increased activation (*p-FWE* < 0.05) in the bilateral amygdala, occipitotemporal cortex (with strongest activation in the fusiform face area), parietal cortex, lateral prefrontal cortex, and the cerebellum.

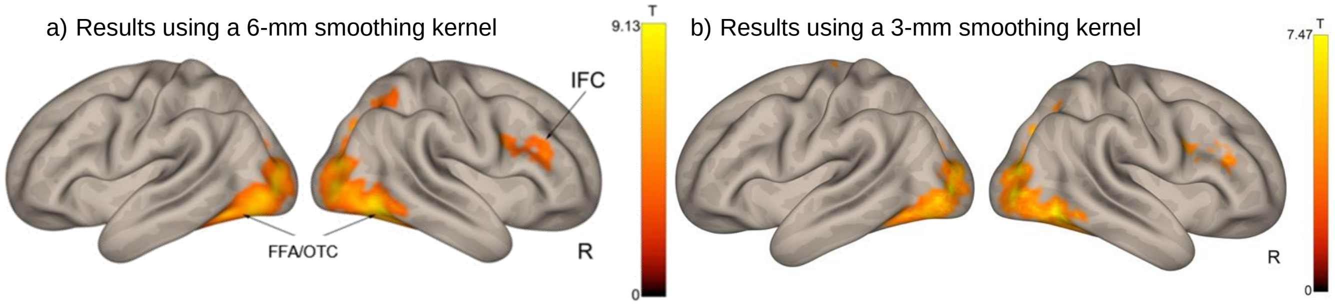

Fig S3. Whole amygdala connectivity pattern in response to Faces (vs. Shapes) contrast using a) 6-mm and b) 3-mm smoothing kernels.

There were consistent findings when using a 6-mm and a 3-mm smoothing kernel. In both analyses, the amygdala was connected to a network including the including bilateral occipitotemporal and parietal cortices and right lateral prefrontal cortex (*p-FWE* < 0.05, cluster corrected). In addition, the area with strongest connectivity in both analyses was the right fusiform face area (FFA).

EFMT

The pattern of scores in eight participants indicated that they held the button box upside down. Their scores were therefore reversed. Overall, task performance on EFMT was close to ceiling, with participants making on average 96.89% (±*SEM* = 0.38) correct responses across all conditions. *Fig. S4* displays the means and standard errors for accuracy (i.e., percentage correct) and reaction times (milliseconds) for all conditions (Fear, Angry, Happy and Shapes). For accuracy, a significant main effect was evident for the Condition effect [$F(3,255) = 11.84$, $p < 0.001$, *Table S3*] with highest-to-lowest accuracy in the following pattern: Shapes>Happy>Fear>Angry. A significant effect of Condition [$F(3,255) = 293.95$, $p < 0.001$, *Table S1*] was also observed for response times with the following fastest-to-slowest pattern: Shapes>Happy>Fear>Angry.

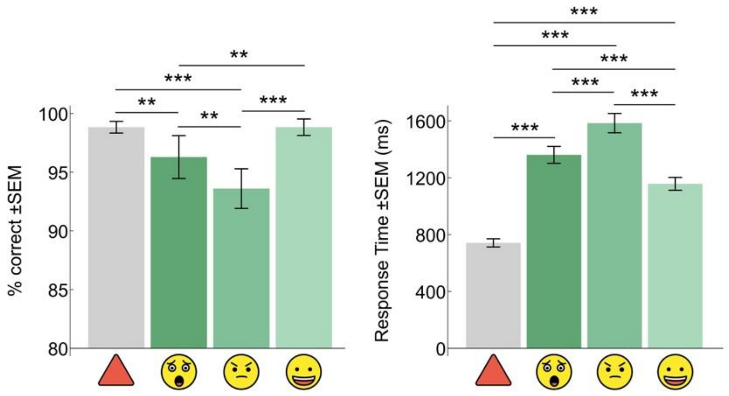

Fig. S4. Accuracy (percentage correct) and response times (RT) in the emotional face matching task. 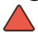 (Shapes), 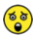 (Fear), 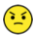 (Anger), and 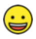 (Happy) conditions. Statistically significant differences between conditions are indicated by * and —— over the relevant conditions; *** *p-FDR* < 0.001; ** *p-FDR* < 0.01).

Table S1.1. Whole amygdala *– Mixed effects models investigating the effect of Emotion on the BOLD response, together with Sex and Laterality effects*

Main effects					
		F		***p***	
Emotion		$F(2,420) = .638$		0.529	
Sex		$F(1,83) = 7.022$		0.010	
Side		$F(1,420) = 22.499$		<0.001	
Simple effects of Emotion					
		**Beta**	***Z***	***p***	***p-FDR***[1]
Fear		0.364	10.394	<0.001	<0.001
Anger		0.351	9.841	<0.001	<0.001
Happy		0.393	12.603	<0.001	<0.001
Sex effect at Emotion					
		F		***p***	***p-FDR***[1]
Fear		$F(1,83) = 5.816$		0.018	0.054
Angry		$F(1,83) = 1.543$		0.218	0.218
Happy		$F(1,83) = 4.212$		0.043	0.065
Effect of Female and Male at Emotion					
		**Beta**	***Z***	***p***	***p-FDR***[2]
Fear:	Female	0.278	6.755	<0.001	<0.001
	Male	0.453	7.744	<0.001	<0.001
Anger:	Female	0.309	7.534	<0.001	<0.001
	Male	0.395	6.925	<0.001	<0.001
Happy:	Female	0.326	9.265	<0.001	<0.001
	Male	0.463	8.500	<0.001	<0.001
Laterality effect at Emotion					
		F		***p***	***p-FDR***[1]
Fear		$F(1,91) = 13.541$		<0.001	0.001
Angry		$F(1,91) = 13.006$		0.001	0.001
Happy		$F(1,91) = 12.202$		0.001	0.001
Effect of Left and Right at Emotion					
		**Beta**	***Z***	***p***	***p-FDR***[2]
Fear:	Left	0.311	9.676	<0.001	<0.001

	Right	0.417	9.737	<0.001	<0.001
Anger:	Left	0.312	9.229	<0.001	<0.001
	Right	0.390	9.647	<0.001	<0.001
Happy:	Left	0.347	10.811	<0.001	<0.001
	Right	0.439	12.360	0.004	0.004

p, uncorrected p-value; *p-FDR*, p-value FDR corrected for multiple comparisons at *p* = 0.05.

[1] *p-FDR* corrected for three emotions

[2] *p-FDR* corrected for six pairwise comparisons

***Table S1.2. Amygdalostriatal** – Mixed effects models investigating the effect of Emotion on the BOLD response, together with Sex and Laterality effects.*

Main effects					
		F		***p***	***p-FDR*[1]**
Emotion		F(2,420) = 1.192		0.305	0.609
Sex		F(1,83) = 3.269		0.074	0.074
Side		F(1,420) = 13.632		<0.001	0.001
Simple effects of Emotion					
		**Beta**	***Z***	***p***	***p-FDR*[2]**
Fear		0.259	10.189	<0.001	<0.001
Anger		0.252	9.327	<0.001	<0.001
Happy		0.293	11.772	<0.001	<0.001
Sex effect at Emotion					
		F		***p***	***p-FDR*[2]**
Fear		F(1,83) = 3.479		0.066	0.197
Angry		F(1,83) = 1.162		0.284	0.426
Happy		F(1,83) = 0.954		0.332	0.332
Effect of Female and Male at Emotion					
		**Beta**	***Z***	***p***	***p-FDR*[3]**
Fear:	Female	0.210	7.736	<0.001	<0.001
	Male	0.311	6.894	<0.001	<0.001
Anger:	Female	0.224	6.690	<0.001	<0.001
	Male	0.281	6.766	<0.001	<0.001
Happy:	Female	0.268	7.892	<0.001	<0.001
	Male	0.320	8.306	<0.001	<0.001
Laterality effect at Emotion					
		F		***p***	***p-FDR*[2]**
Fear		F(1,91) = 7.398		0.008	0.012
Angry		F(1,91) = 2.071		0.154	0.154
Happy		F(1,91) = 13.222		<0.001	0.001
Effect of Left and Right at Emotion					
		**Beta**	***Z***	***p***	***p-FDR*[3]**
Fear:	Left	0.223	8.716	<0.001	<0.001

	Right	0.296	9.323	<0.001	<0.001
Anger:	Left	0.233	8.354	<0.001	<0.001
	Right	0.271	8.461	<0.001	<0.001
Happy:	Left	0.244	10.003	<0.001	<0.001
	Right	0.342	10.789	<0.001	<0.001

p, uncorrected p-value; *p-FDR*, p-value FDR corrected for multiple comparisons at $p = 0.05$.

[1] *p-FDR* corrected for four subregions

[2] *p-FDR* corrected for three emotions

[3] *p-FDR* corrected for six post-hoc tests

Table S1.3. Centromedial *– Mixed effects models investigating the effect of Emotion on the BOLD response, together with Sex and Laterality effects*

Main effects				
	F		***p***	***p-FDR*[1]**
Emotion	F(2,420) = 0.201		0.818	1.000
Sex	F(1,83) = 10.286		0.002	0.008
Side	F(1,420) = 8.712		0.003	0.003
Simple effects of Emotion				
	**Beta**	***Z***	***p***	***p-FDR*[2]**
Fear	0.495	12.065	<0.001	<0.001
Anger	0.466	10.793	<0.001	<0.001
Happy	0.492	12.963	<0.001	<0.001
Sex effect at Emotion				
	F		***p***	***p-FDR*[2]**
Fear	F(1,83) = 7.126		0.009	0.027
Angry	F(1,83) = 3.471		0.066	0.066
Happy	F(1,83) = 6.3041		0.014	0.021
Effect of Female and Male at Emotion				
	**Beta**	***Z***	***p***	***p-FDR*[3]**
Fear: Female	0.383	7.611	<0.001	<0.001
Male	0.611	9.076	<0.001	<0.001
Anger: Female	0.390	7.733	<0.001	<0.001
Male	0.546	7.916	<0.001	<0.001
Happy: Female	0.393	8.440	<0.001	<0.001
Male	0.597	9.377	<0.001	<0.001
Laterality effect at Emotion				
	F		***p***	***p-FDR*[2]**
Fear	F(1,91) = 6.653		0.011	0.034
Angry	F(1,91) = 3.831		0.053	0.080
Happy	F(1,91) = 3.710		0.054	0.054
Effect of Left and Right at Emotion				
	**Beta**	***Z***	***p***	***p-FDR*[3]**
Fear: Left	0.453	11.631	<0.001	<0.001

	Right	0.536	11.006	<0.001	<0.001
Anger:	Left	0.440	10.106	<0.001	<0.001
	Right	0.492	10.482	<0.001	<0.001
Happy:	Left	0.467	11.950	<0.001	<0.001
	Right	0.518	12.530	<0.001	<0.001

p, uncorrected p-value; *p-FDR*, p-value FDR corrected for multiple comparisons at $p = 0.05$.

[1] *p-FDR* corrected for four subregions

[2] *p-FDR* corrected for three emotions

[3] *p-FDR* corrected for six post-hoc tests

Table S1.4. Basolateral *– Mixed effects models investigating the effect of Emotion on the BOLD response, together with Sex and Laterality effects*

Main effects					
		F		***p***	***p-FDR*[1]**
Emotion		F(2,420) = 1.245		0.289	1.000
Sex		F(1,83) = 5.102		0.027	0.035
Side		F(1,420) = 13.548		<0.001	0.001
Simple effects of Emotion					
		**Beta**	***Z***	***p***	***p-FDR*[2]**
Fear		0.286	8.794	<0.001	<0.001
Anger		0.271	8.493	<0.001	<0.001
Happy		0.325	11.634	<0.001	<0.001
Sex effect at Emotion					
		F		***p***	***p-FDR*[2]**
Fear		F(1,83) = 5.398		0.023	0.068
Angry		F(1,83) = 0.725		0.397	0.397
Happy		F(1,83) = 2.738		0.102	0.153
Effect of Female and Male at Emotion					
		**Beta**	***Z***	***p***	***p-FDR*[3]**
Fear:	Female	0.211	5.503	<0.001	<0.001
	Male	0.365	6.799	<0.001	<0.001
Anger:	Female	0.246	6.848	<0.001	<0.001
	Male	0.298	5.788	<0.001	<0.001
Happy:	Female	0.277	9.014	<0.001	<0.001
	Male	0.375	7.680	<0.001	<0.001
Laterality effect at Emotion					
		F		***p***	***p-FDR*[2]**
Fear		F(1,91) = 6.992		0.010	0.014
Angry		F(1,91) = 8.312		0.005	0.015
Happy		F(1,91) = 6.540		0.012	0.012
Effect of Left and Right at Emotion					
		**Beta**	***Z***	***p***	***p-FDR*[3]**

Fear:	Left	0.244	7.793	<0.001	<0.001
	Right	0.328	8.101	<0.001	<0.001
Anger:	Left	0.237	7.714	<0.001	<0.001
	Right	0.305	8.249	<0.001	<0.001
Happy:	Left	0.287	9.451	<0.001	<0.001
	Right	0.363	11.028	<0.001	<0.001

p, uncorrected p-value; *p-FDR*, p-value FDR corrected for multiple comparisons at $p = 0.05$.

[1] *p-FDR* corrected for four subregions

[2] *p-FDR* corrected for three emotions

[3] *p-FDR* corrected for six post-hoc tests

Table S1.5. Superficial *– Mixed effects models investigating the effect of Emotion on the BOLD response, together with Sex and Laterality effects*

Main effects					
		F		***p***	***p-FDR[1]***
Emotion		F(2,420) = 0.046		0.955	0.955
Sex		F(1,83) = 6.844		0.011	0.021
Side		F(1,420) = 9.893		0.002	0.002
Simple effects of Emotion					
		**Beta**	***Z***	***p***	***p-FDR[2]***
Fear		0.618	9.870	<0.001	<0.001
Anger		0.636	10.141	<0.001	<0.001
Happy		0.639	11.234	<0.001	<0.001
Sex effect at Emotion					
		F		***p***	***p-FDR[2]***
Fear		F(1,83) = 3.204		0.077	0.116
Angry		F(1,83) = 2.287		0.134	0.134
Happy		F(1,83) = 5.456		0.022	0.066
Effect of Female and Male at Emotion					
		**Beta**	***Z***	***p***	***p-FDR[3]***
Fear:	Female	0.504	6.442	<0.001	<0.001
	Male	0.738	7.232	<0.001	<0.001
Anger:	Female	0.546	7.452	<0.001	<0.001
	Male	0.730	7.299	<0.001	<0.001
Happy:	Female	0.501	8.108	<0.001	<0.001
	Male	0.783	7.793	<0.001	<0.001
Laterality effect at Emotion					
				p	***p-FDR[2]***
Fear		F(1,91) = 12.535		0.001	0.002
Angry		F(1,91) = 3.148		0.079	0.079
Happy		F(1,91) = 5.337		0.023	0.035
Effect of Left and Right at Emotion					
		**Beta**	***Z***	***p***	***p-FDR[3]***
Fear:	Left	0.532	8.308	<0.001	<0.001

	Right	0.705	10.021	<0.001	<0.001
Anger:	Left	0.596	8.834	<0.001	<0.001
	Right	0.677	10.274	<0.001	<0.001
Happy:	Left	0.580	8.776	<0.001	<0.001
	Right	0.698	11.972	<0.001	<0.001

Astr., amygdalostriatal; CM, centromedial; BL, basolateral; SF, superficial; *p*, uncorrected p-value; *p-FDR*, p-value FDR corrected for multiple comparisons at p = 0.05.

[1] *p-FDR* corrected for four subregions

[2] *p-FDR* corrected for three emotions

[3] *p-FDR* corrected for six post-hoc tests

Table S1.6. Subregions *– Mixed effect models investigating the effect of Region on the BOLD response, together with Sex and Hemisphere effects.*

		**Beta**	***Z***	***p***	***p-FDR*[1]**
Simple effects of Fear					
Astr.		0.259	10.010	<0.001	<0.001
CM		0.495	12.050	<0.001	<0.001
BL		0.286	8.777	<0.001	<0.001
SF		0.618	9.859	<0.001	<0.001
Simple effects of Anger					
**Contrast**		**Beta**	***Z***	***p***	***p-FDR*[1]**
Astr.		0.252	8.995	<0.001	<0.001
CM		0.466	10.688	<0.001	<0.001
BL		0.271	8.395	<0.001	<0.001
SF		0.636	10.126	<0.001	<0.001
Simple effects of Happy					
**Contrast**		**Beta**	***Z***	***p***	***p-FDR*[1]**
Astr.		0.293	11.666	<0.001	<0.001
CM		0.492	12.966	<0.001	<0.001
BL		0.325	11.642	<0.001	<0.001
SF		0.639	11.294	<0.001	<0.001
Pairwise differences between subregions					
**Contrast**		**Beta**	***Z***	***p***	***p-FDR*[2]**
Fear:	SF>Astr.	0.360	6.998	<0.001	<0.001
	SF>CM	0.124	3.669	<0.001	<0.001
	SF>BL	0.333	8.091	<0.001	<0.001
	CM>Astr.	0.237	8.735	<0.001	<0.001
	CM>BL	0.210	7.927	<0.001	<0.001
	BL>Astr.	0.027	1.141	0.254	0.254
Anger:	SF>Astr.	0.386	8.376	<0.001	<0.001
	SF>CM	0.170	5.652	<0.001	<0.001
	SF>BL	0.367	9.438	<0.001	<0.001
	CM>Astr.	0.215	9.038	<0.001	<0.001
	CM>BL	0.196	7.794	<0.001	<0.001
	BL>Astr.	0.019	0.857	0.391	0.391

Happy:	SF>Astr.	0.348	8.218	<0.001	<0.001
	SF>CM	0.147	4.982	<0.001	<0.001
	SF>BL	0.316	8.755	<0.001	<0.001
	CM>Astr.	0.201	10.064	<0.001	<0.001
	CM>BL	0.169	7.092	<0.001	<0.001
	BL>Astr.	0.032	1.647	0.100	0.100

Astr., amygdalostriatal; CM, centromedial; BL, basolateral; SF, superficial; *p*, uncorrected p-value; *p-FDR*, p-value FDR corrected for multiple comparisons at $p = 0.05$.

[1] *p-FDR* corrected for four subregions

[2] *p-FDR* corrected for six pairwise comparisons

Table S2.1. Whole amygdala connectivity *– gPPI analysis with selected ROIs*

Pairwise differences in amygdala connectivity between Emotions						
		**Contrast**	**Beta**	***T***	***p***	***p-FDR*[1]**
Right FFA		Fear>Anger	0.03	1.70	0.093	0.108
		Fear>Happy	0.04	2.68	0.009	0.035
		Anger>Happy	0.01	0.89	0.374	0.517
Left FFA		Fear>Anger	0.03	1.96	0.054	0.107
		Fear>Happy	0.02	1.29	0.201	0.268
		Anger>Happy	-0.01	-0.47	0.637	0.637
Right IFC		Fear>Anger	0.04	2.75	0.007	0.029
		Fear>Happy	0.03	1.86	0.066	0.133
		Anger>Happy	-0.01	-0.87	0.388	0.517
Left Cerebellum		Fear>Anger	0.01	1.62	0.108	0.108
		Fear>Happy	-0.01	-1.07	0.286	0.517
		Anger>Happy	0.00	0.53	0.601	0.601
Effect of Sex[†]						
		**Contrast**	**Beta**	***T***	***p*[2]**	
Fear:	Right FFA	Female>Male	0.02	0.97	0.337	
	Right IFC	Female>Male	-0.04	-1.49	0.139	
Anger:	Right FFA	Female>Male	0.02	0.80	0.427	
	Right IFC	Female>Male	0.02	0.68	0.499	
Happy:	Right FFA	Female>Male	-0.02	-0.58	0.562	
	Right IFC	Female>Male	0.00	0.13	0.901	
Laterality effect[†]						
		**Contrast**	**Beta**	***T***	***p***	***p-FDR*[3]**
Fear:	Right FFA	Right>Left	0.03	1.79	0.076	0.153
	Right IFC	Right>Left	-0.01	-0.38	0.702	0.702
Anger:	Right FFA	Right>Left	0.01	0.42	0.675	0.675
	Right IFC	Right>Left	-0.03	-2.14	0.035	0.070
Happy:	Right FFA	Right>Left	0.00	0.09	0.930	0.930
	Right IFC	Right>Left	0.00	-0.12	0.901	0.930

FFA, fusiform face area; IFC, inferior frontal cortex; *p*, uncorrected p-value; *p-FDR*, p-value FDR corrected for multiple comparisons at $p = 0.05$.

[1] *p-FDR* corrected for 12 comparisons

[2] No correction for multiple comparisons as there were no significant uncorrected results

[3] *p-FDR* corrected for six comparisons

[†] Restricted to Right FFA and IFC as these regions exhibited an Emotion effect

***Table S2.2. Amygdala subregions connectivity** – gPPI analysis with selected ROIs*[†]

		**Beta**	***T***	***p***	***p-FDR***[1]
Fear					
Astr.	Right FFA	0.04	3.95	<0.001	<0.001
	Right IFC	0.01	0.99	0.324	0.324
CM	Right FFA	0.05	4.31	<0.001	<0.001
	Right IFC	0.05	5.28	<0.001	<0.001
BL	Right FFA	0.14	6.57	<0.001	<0.001
	Right IFC	0.09	4.29	<0.001	<0.001
SF	Right FFA	0.07	6.55	<0.001	<0.001
	Right IFC	0.04	4.40	<0.001	<0.001
Anger					
Astr.	Right FFA	0.05	4.57	<0.001	<0.001
	Right IFC	0.01	1.22	0.225	0.225
CM	Right FFA	0.03	2.86	0.005	0.011
	Right IFC	0.02	1.82	0.072	0.072
BL	Right FFA	0.10	4.72	<0.001	<0.001
	Right IFC	0.02	1.26	0.211	0.211
SF	Right FFA	0.04	4.64	<0.001	<0.001
	Right IFC	0.02	1.60	0.113	0.113
Happy					
Astr.	Right FFA	0.02	1.92	0.058	0.096
	Right IFC	0.02	1.68	0.096	0.096
CM	Right FFA	0.03	2.54	0.013	0.019
	Right IFC	0.03	2.39	0.019	0.019
BL	Right FFA	0.07	3.29	0.001	0.003
	Right IFC	0.05	2.67	0.009	0.009
SF	Right FFA	0.04	3.89	<0.001	<0.001
	Right IFC	0.02	3.06	0.003	0.003

FFA, fusiform face area; IFC, inferior frontal cortex; Astr., amygdalostriatal; CM, centromedial; BL, basolateral; SF, superficial; *p*, uncorrected p-value; *p-FDR*, p-value FDR corrected for multiple comparisons at p = 0.05.

[1] *p-FDR* corrected for eight tests

[†] Restricted to Right FFA and IFC as these regions exhibited an Emotion effect

***Table S2.3. Basolateral (LB) connectivity** – Statistical comparisons to all other amygdala subregions† (Amygalostriatal [Astr], Centromedial [CM], Superficial [SF]) for the individual emotions (Fear, Anger, Happy)*

	Contrast	Beta	*T*	*p*	*p-FDR*[1]
Fear					
Right FFA	BL>Astr	0.05	5.04	<0.001	<0.001
	BL>CM	0.05	4.60	<0.001	<0.001
	BL>SF	0.03	3.15	0.002	0.005
Right IFC	BL>Astr	0.04	3.92	<0.001	<0.001
	BL>CM	0.02	1.89	0.062	0.062
	BL>SF	0.02	2.33	0.022	0.022
Anger					
Right FFA	BL>Astr	0.03	2.46	0.016	0.032
	BL>CM	0.03	3.34	0.001	0.003
	BL>SF	0.03	2.89	0.005	0.010
Right IFC	BL>Astr	0.01	0.56	0.576	0.576
	BL>CM	0.00	0.08	0.937	0.937
	BL>SF	0.00	0.41	0.686	0.686
Happy					
Right FFA	BL>Astr	0.03	2.40	0.018	0.037
	BL>CM	0.02	2.14	0.035	0.070
	BL>SF	0.02	1.91	0.059	0.119
Right IFC	BL>Astr	0.02	1.71	0.092	0.092
	BL>CM	0.01	1.31	0.193	0.193
	BL>SF	0.01	1.50	0.138	0.138

FFA, fusiform face area; IFC, inferior frontal cortex; *p*, uncorrected p-value; *p-FDR*, p-value FDR corrected for multiple comparisons at p = 0.05.

[1] *p-FDR* corrected for six comparisons

† Restricted to Right FFA and IFC as these regions exhibited an Emotion effect

Table S3. Behavioural data *– Mixed effect models (pairwise comparisons between conditions) comparing behavioural performance (percent correct and response time) on the Emotional Face Matching Task.*

	**Beta**	***Z***	***p***	***p-FDR*[1]**
Accuracy				
Fear vs. Shapes	-2.543	-2.535	0.011	0.014
Anger vs. Shapes	-5.232	-5.834	<0.001	<0.001
Happy vs. Shapes	0.001	0.002	0.998	0.998
Anger vs. Fear	-2.689	-3.111	0.002	0.004
Happy vs. Fear	2.544	2.739	0.006	0.009
Happy vs. Anger	5.233	5.470	<0.001	<0.001
Response time				
Fear vs. Shapes	619.627	23.715	<0.001	<0.001
Anger vs. Shapes	843.201	27.350	<0.001	<0.001
Happy vs. Shapes	415.973	23.994	<0.001	<0.001
Anger vs. Fear	223.574	11.422	<0.001	<0.001
Happy vs. Fear	-203.654	-9.957	<0.001	<0.001
Happy vs. Anger	-427.228	-16.154	<0.001	<0.001

p, uncorrected p-value; *p-FDR*, p-value FDR corrected for multiple comparisons at p = 0.05.

[1] *p-FDR* corrected for six comparisons
